# Potassium intake, skeletal muscle mass, and effect modification by sex: data from the 2008–2011 KNHANES

**DOI:** 10.1186/s12937-020-00614-z

**Published:** 2020-08-29

**Authors:** Yu-Ji Lee, Mirae Lee, Yu Mi Wi, Seong Cho, Sung Rok Kim

**Affiliations:** Department of Internal Medicine, Samsung Changwon Hospital, Sungkyunkwan University School of Medicine, 158, Paryong-ro, Masanhoewon-gu, 51353 Changwon, Republic of Korea

**Keywords:** Dietary potassium, Skeletal muscle, Sex-specific difference

## Abstract

**Background:**

A loss of muscle mass may be influenced by multiple factors. Insulin sensitivity and metabolic acidosis are associated with muscle wasting and may be improved with potassium intake. This study evaluated the association between dietary potassium intake and skeletal muscle mass.

**Methods:**

We performed a cross-sectional study with data obtained from the Korean National Health and Nutrition Examination Survey (KNHANES) (2008–2011). Participant’s daily food intake was assessed using a 24-h recall method. Appendicular skeletal muscle mass (ASM) was calculated as the sum of muscle mass in both arms and legs, measured using dual energy X-ray absorptiometry. The skeletal muscle index (SMI) was calculated as ASM divided by height^2^ (kg/m^2^). Low muscle mass was defined as a SMI < 7.0 kg/m^2^ for men and < 5.4 kg/m^2^ for women.

**Results:**

Data from 16,558 participants (age ≥ 19 years) were analyzed. Participants were categorized into quintiles according to their potassium intake. Sex-specific differences were found in the association between potassium intake and muscle mass (*P*_Interaction_ < 0.001). In men, higher potassium intake was associated with lower odds for low muscle mass; the fully adjusted odds ratios (95% confidence intervals) were 0.78 (0.60–1.03), 0.71 (0.54–0.93), 0.68 (0.51–0.90), and 0.71 (0.51–0.98) for the top four quintiles (referenced against the lowest quintile), respectively. However, this association was attenuated in women after adjusting for total energy intake. Higher potassium intakes were also associated with a greater SMI.

**Conclusions:**

Higher dietary potassium intake decreased the odds of low muscle mass in men but not in women.

## Introduction

Skeletal muscle mass is a body tissue that play an important role in metabolic regulation, movement, and strength [1, 2]. Muscle mass decreases as much as 3–8% each decade after the age of 30–40 years and the rate of loss increases to 15% each decade after the age of 70 years [3]. A loss of muscle mass and strength resulting in impaired physical function, a condition called sarcopenia, is now recognized as a clinical concern for public health, especially in the aging population [4, 5]. Low muscle mass leads to reduced muscle function, [6] which may be associated with an increased risk of adverse health outcomes such as type 2 diabetes, metabolic syndrome, cardiovascular disease, frailty, and reduced quality of life [7–10]. Therefore, for overall health it is important to maintain optimal muscle mass across the lifespan.

Although a loss of muscle mass usually occurs with aging, the degree or rate of decrease in muscle mass varies across the population, indicating that the loss of muscle mass may be influenced by multiple factors [11]. In particular, loss of muscle mass is thought to be faster in men than in women, which may be mainly mediated by hormonal factors [12]. In addition, lack of physical activity, malnutrition, chronic diseases, insulin resistance or mild metabolic acidosis can be associated with muscle wasting [13–16].

Potassium is a vital element that plays an important role in maintaining cell function, particularly in muscles and nerves and has various health benefits; increased potassium intake has proved to be effective in reducing blood pressure [17] and was associated with a reduced risk of stroke [18]. As a result, the World Health Organization recommends an increase in potassium intake from food for reducing blood pressure and the risks of cardiovascular disease and stroke in adults [19]. Dietary potassium intake may also have a beneficial effect on bone health and insulin sensitivity [20, 21]. Higher potassium intake was associated with enhanced bone mineral density, probably by neutralizing acid load and consequently reducing calcium loss from the bone [20, 22, 23]. Lower potassium intake was associated with lower insulin sensitivity [21, 24].

The proposed methods to prevent a loss of muscle mass in the elderly are mainly protein intake and exercise [25, 26]. However, there have been little evidence of the effectiveness of other dietary approaches on muscle mass. Although some studies show an association between the intake of minerals, such as calcium and magnesium, and muscle mass, there are few studies on the role of potassium intake on muscle mass [27–29]. Given any association between factors that may be associated with muscle loss, such as insulin resistance or mild metabolic acidosis, and the beneficial effects of potassium on these factors, we sought to examine the association between dietary potassium intake and muscle mass. Furthermore, given that loss of muscle mass is different between men and women, we sought to evaluate the association of dietary potassium intake with loss of muscle mass according to sex.

## Materials and methods

### Study subjects

We extracted and examined data obtained from the fourth and fifth Korean National Health and Nutrition Examination Survey (KNHANES), conducted by the Korean Centers for Disease Control and Prevention (KCDC) from 2008 to 2011. This survey used a stratified, multistage clustered probability sampling method to collect a representative sample of the noninstitutionalized Korean population. A total of 9744; 10,533; 8958; and 8518 participants were recruited in KNHANES in 2008, 2009, 2010, and 2011, respectively, and the response rates were 77.8, 82.8, 81.9, and 80.4%, respectively. The survey was composed of a health interview, health examination, and nutrition survey.

All participants signed a written informed consent form and the protocols for KNHANES were approved by the Institutional review board of the KCDC. The KNHANES database is publicly available at the KNHANES website (http://knhanes.cdc.go.kr).

### Demographic and laboratory measurements

Demographic factors (age and sex), history of diabetes and hypertension, and smoking status (current, past, or never) were obtained from the datasets. Blood samples were collected from participants after they had fasted for 8 h. Samples were then transported to a central laboratory (Neodin Medical Institute, Seoul, South Korea) and analyzed within 24 h. White blood cell count (WBC), serum hemoglobin, fasting glucose, total cholesterol, and serum creatinine (sCr) values were used for analyses. Estimated glomerular filtration rate (eGFR) was calculated using the Chronic Kidney Disease Epidemiology Collaboration equation [30] as follows:

eGFR (mL/min/1.73 m^2^) = 141 × min (sCr/κ, 1)^α^ × max (sCr/κ, 1)^–1.209^ × 0.993^Age^ (× 1.018 if female) (× 1.159 if black), where κ is 0.7 for women and 0.9 for men, α is − 0.329 for women and − 0.411 for men, min (sCr/κ, 1) is the minimum of sCr/κ or 1, and max (sCr/κ, 1) is the maximum of sCr/κ or 1.

### Anthropometric measurement and assessment of physical activity

Height and weight were measured directly by a trained investigator using a stadiometer and scale while the participants were wearing light clothing without shoes. Body mass index (BMI) was calculated as the individual’s weight (in kg) divided by their height (in meters squared) (kg/m^2^). Physical activity was evaluated by asking participants their physical activity level during each week using the Korean version of the International Physical Activity Questionnaire [31]. Physical activity levels were categorized as high, moderate, or low. High physical activity was defined as ≥20 min at a time of vigorous-intensity activity on at least 3 days per week. Moderate physical activity was defined as ≥30 min at a time of moderate-intensity activity on at least 5 days per week. Other physical activity was defined as all remaining intensities and volumes of activity.

### Assessment of dietary nutrient intake

Dietary intake was surveyed in a face-to-face interview at the participant’s home. The food intake questionnaire is an open-ended survey using a 24-h recall method with various measuring aids to determine the participant’s intake of various dishes and foods. Survey staff members completed an intensive training course and conducted supervised practice interviews before conducting a survey with a participant. Survey staff were retrained five to six times a year to ensure they followed proper techniques and protocols. The types and quantities of food examined were coded by computerized methods and nutrient intake levels were determined from this data using various databases and formulas. The details of the nutrition survey protocol are described on the KNHANES website (http://knhanes.cdc.go.kr).

### Measurements of skeletal muscle mass index

Bone mineral contents and body composition were measured by licensed technicians using dual energy X-ray absorptiometry (DXA) (Discovery QDR 4500 W, Hologic Inc., Belford, MA, USA). Participants fasted prior to the assessment and were in the supine position during the assessment. All non-fat and non-bone tissue was assumed to be skeletal muscle. Appendicular skeletal muscle mass (ASM) was calculated as the sum of skeletal muscle mass in both arms and legs, as measured by DXA. The subject’s skeletal muscle mass index (SMI) was calculated as their ASM (kg) divided by their height in meters squared (m^2^). Low muscle mass was defined as SMI values < 7.0 kg/m^2^ for men and < 5.4 kg/m^2^ for women, as recommended by the Asian Working Group for Sarcopenia [32].

### Statistical methods

Characteristics of participants were described across potassium intake quintiles and trends across categories were evaluated using either a Wilcoxon-type non-parametric trend test or a linear regression model, as appropriate. The association between categorized potassium intake and low muscle mass was assessed with logistic regression models, using the lowest quintile as a reference. Four hierarchical models were examined: (1) adjusted for age and sex (Model 1); (2) adjusted for additional comorbidities (diabetes and hypertension), BMI, smoking status, number of days of weight training per week, physical activity (high, moderate, or other), WBC, hemoglobin, and total cholesterol (Model 2); (3) adjusted for the variables included in Model 2, eGFR, and protein intake (Model 3); and (4) adjusted for the variables included in Model 3 and total energy intake (Model 4). Effect modification by sex was examined by adding the interaction terms into the logistic regression models, followed by subgroup analyses (man or woman). A receiver operating characteristic (ROC) curve was constructed to assess the ability of dietary potassium intake to predict low muscle mass. The association between potassium intake, as a continuous variable, and low muscle mass, adjusted as previously mentioned, was examined. A restricted cubic spline model with four knots was also used to examine the association of potassium intake with low muscle mass. In sensitivity analysis, the association between potassium intake and SMI was assessed using a linear regression model with the four aforementioned adjustment levels.

We assessed missing data in our covariates and found that hypertension, BMI, smoking status, number of days of weight training per week, physical activity, WBC, hemoglobin, cholesterol, and protein intake had missing data in < 5% of our cohort, except for diabetes (6.8%). We performed multiple imputations using a multivariate normal distribution. We included the variables including age, sex, hypertension, diabetes, BMI, smoking status, number of days of weight training per week, physical activity, WBC, hemoglobin, cholesterol, eGFR, protein intake, total energy intake, and SMI in the imputation model using 10 imputed datasets. The estimates were combined using Rubin’s rules [33]. Statistical analyses were conducted using STATA, version 13.1 (StataCorp LP, College Station, TX, USA).

## Results

### Patient characteristics

**Fig. 1:**
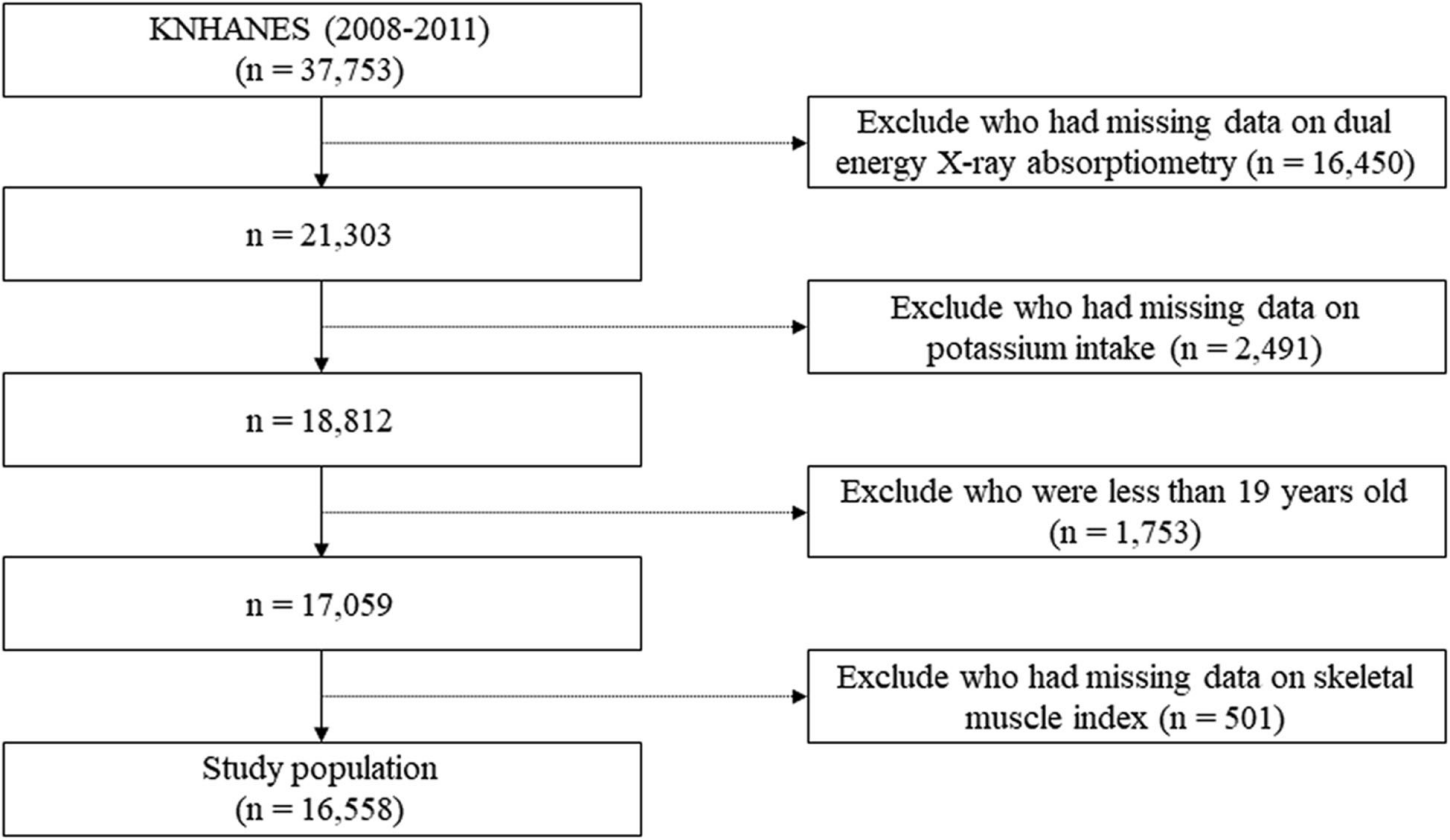
Participant flow diagram

Among the 37,753 participants included in the KNHANES, the 17,059 subjects aged ≥19 years whose surveys included DXA and 24-h dietary recall data were included in this cross-sectional study. Of those potential subjects, 501 subjects without data on SMI were excluded from the analysis (Fig. 1). Thus, a total of 16,558 participants were finally analyzed in this study. The participants were categorized into quintiles according to their daily potassium intake; the median (interquartile range [IQR]) potassium intake of each quintile was 1.4 (1.1–1.6), 2.1 (1.9–2.2), 2.7 (2.5–2.9), 3.5 (3.2–3.7), and 4.9 (4.4–5.8) g/day. The baseline characteristics of the study participants across quintiles of potassium intake are described in Table 1. In the overall cohort, the mean age was 50 ± 16 years and 40% were men. Median (IQR) potassium intake was 2.7 (1.9–3.7) g/day. Median (IQR) SMI was 6.6 kg/m^2^. Mean ± SD baseline eGFR was 96 ± 17 mL/min/1.73 m^2^. The proportion of participants with eGFR < 30 mL/min/1.73 m^2^ was less than 0.2%. Subjects with higher potassium intakes tended to be men. Subjects with higher potassium intakes also had a larger SMI, higher protein intake, and lower prevalence of hypertension.

**Table 1:** Baseline characteristics of 16,558 participants according to baseline potassium intake quintiles

		Dietary Potassium Intake					
	Total	Quintile 1	Quintile 2	Quintile 3	Quintile 4	Quintile 5	*P* value
N (%)	16,558	3311 (20)	3312 (20)	3311 (20)	3312 (20)	3312 (20)	
Age, years	50 ± 16	54 ± 19	50 ± 17	49 ± 16	48 ± 15	48 ± 14	< 0.001
Male, %	40	23	32	41	48	59	< 0.001
Hypertension, %	33	38	33	32	30	30	< 0.001
Diabetes, %	10	12	10	9	9	10	< 0.001
Body weight, kg	62 ± 11	58 ± 11	60 ± 11	62 ± 11	63 ± 11	65 ± 11	< 0.001
Height, cm	162 ± 9	158 ± 9	160 ± 9	162 ± 9	163 ± 9	165 ± 9	< 0.001
Body mass index, kg/m^2^	24 ± 3	23 ± 4	23 ± 3	24 ± 3	24 ± 3	24 ± 3	< 0.001
Weight training, days/week	1.0 (1.0–1.0)	1.0 (1.0–1.0)	1.0 (1.0–1.0)	1.0 (1.0–1.0)	1.0 (1.0–2.0)	1.0 (1.0–3.0)	< 0.001
Physical activity, %							< 0.001
High	15	11	15	14	16	20	
Moderate	9	8	8	8	8	10	
Other	76	81	77	78	76	70	
Current smoker, %	30	25	26	30	33	39	< 0.001
ASM, kg	16.2 (13.8–20.9)	14.6 (12.9–17.2)	15.4 (13.5–19.2)	16.3 (13.9–20.7)	17.4 (14.3–21.9)	19.6 (15.1–23.3)	< 0.001
SMI, kg/m^2^	6.6 ± 1.2	6.2 ± 1.0	6.4 ± 1.1	6.6 ± 1.2	6.8 ± 1.2	7.0 ± 1.2	< 0.001
WBC, cells/μL	6.0 ± 1.7	6.1 ± 1.7	6.0 ± 1.7	6.0 ± 1.6	6.0 ± 1.6	6.1 ± 1.7	0.115
Hemoglobin, g/dL	13.8 ± 1.6	13.4 ± 1.5	13.6 ± 1.5	13.8 ± 1.6	14.0 ± 1.6	14.3 ± 1.5	< 0.001
Total cholesterol, mg/dL	188 ± 36	189 ± 37	188 ± 36	187 ± 36	188 ± 36	188 ± 35	0.357
eGFR, mL/min/1.73 m^2^	96 ± 17	93 ± 19	96 ± 17	97 ± 17	97 ± 16	96 ± 15	< 0.001
Protein intake, g/day	1.0 (0.7–1.4)	0.6 (0.4–0.7)	0.8 (0.7–1.0)	1.0 (0.8–1.3)	1.2 (1.0–1.5)	1.6 (1.2–2.0)	< 0.001
Potassium intake, g/day	2.7 (1.9–3.7)	1.4 (1.1–1.6)	2.1 (1.9–2.2)	2.7 (2.5–2.9)	3.5 (3.2–3.7)	4.9 (4.4–5.8)	< 0.001
Energy intake, kcal/day	1760 (1351–2287)	1133 (874–1389)	1509 (1266–1776)	1767 (1502–2079)	2080 (1746–2477)	2598 (2143–3252)	< 0.001

Note: values for categorical variables are shown as percentages; values for continuous variables, as mean ± standard deviation or median (interquartile range)

Conversion factors for units: to convert hemoglobin from g/dL to g/L multiply by 10; to convert total cholesterol from mg/dL to mmol/L multiply by 0.0259

High physical activity was defined as ≥20 min at a time of vigorous-intensity activity on at least three days per week. Moderate physical activity was defined as ≥30 min at a time of moderate-intensity activity on at least five days per week. Other physical activity was defined as all remaining intensities and volumes of activity

Abbreviations: ASM, appendicular skeletal muscle mass; SMI, skeletal muscle mass index; WBC, white blood cell count; eGFR, estimated glomerular filtration rate

### Association between potassium intake and low muscle mass according to sex

**Fig. 2:**
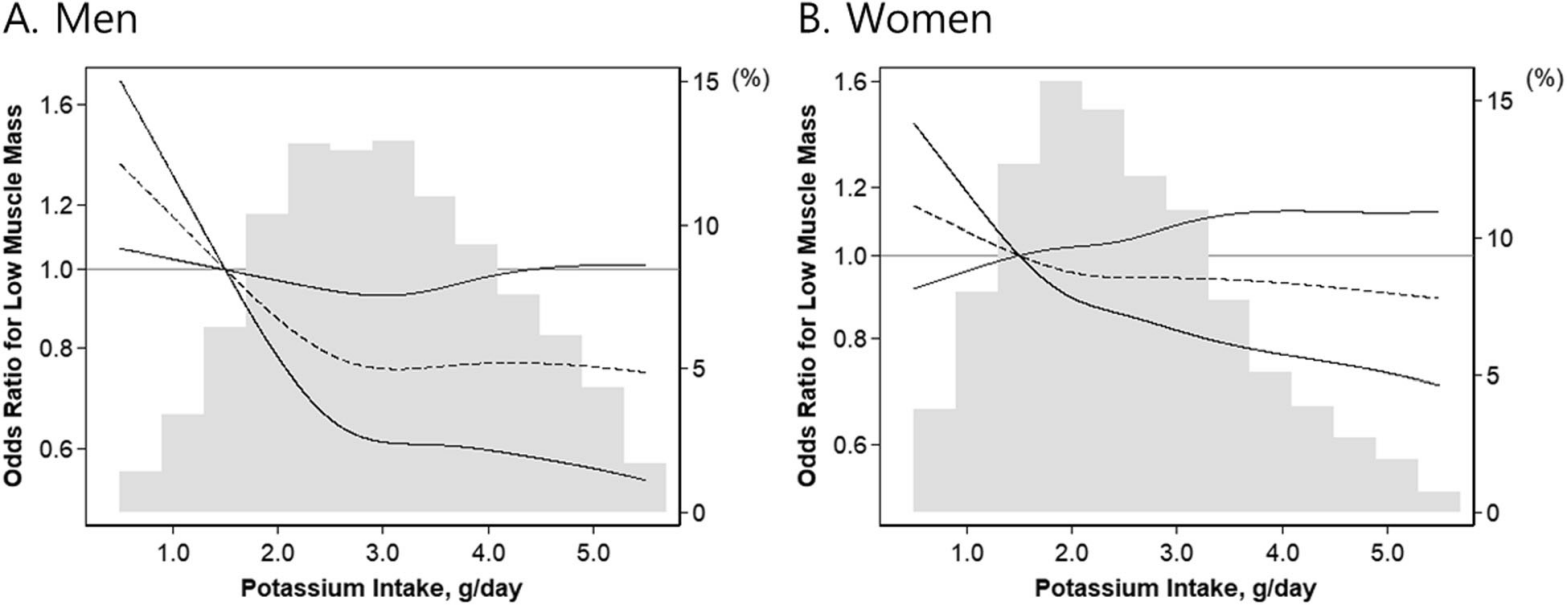
Fully adjusted logistic regression model with a restricted cubic spline for the association between dietary potassium intake as a continuous variable and low muscle mass according to men (**a**) and women (**b**) among 16,558 Korean general population

In our cohort, 4174 (25.2%) subjects met diagnostic criteria for low muscle mass (24% of men and 26% of women). Higher dietary potassium intake tended to be associated with decreased odds for low muscle mass, but the association was attenuated after adjusting for total energy intake (Supplementary Fig. 1). The association between potassium intake and low muscle mass was significantly modified by sex (*P*_Interaction_ < 0.001). For men, higher dietary potassium intake decreased the odds for low muscle mass across all adjustment levels. In a fully adjusted model, the highest quintile of potassium intake was associated with 29% lower odds of low muscle mass compared to the lowest quintile of potassium intake; the fully adjusted odds ratio (OR) and 95% confidence interval (CI) for the second to fifth quintiles were 0.78 (0.60–1.03), 0.71 (0.54–0.93), 0.68 (0.51–0.90), and 0.71 (0.51–0.98). However, for women, the association of higher potassium intake with lower odds for low muscle mass was attenuated after adjusting for total energy intake (Table 2). In restricted cubic spline models, the association between higher potassium intake (as a continuous variable) and low muscle mass similarly showed sex-specific differences; the association was attenuated in women (Fig. 2a and b).

**Table 2:** Odds ratios and 95% confidence intervals for low muscle mass with four levels of adjustment based upon potassium intake quintiles

	Model 1		Model 2		Model 3		Model 4	
	ORs	95% CI	ORs	95% CI	ORs	95% CI	ORs	95% CI
**Men**								
Quintile 1	Reference		Reference		Reference		Reference	
Quintile 2	0.61	0.50–0.74	0.72	0.54–0.94	0.72	0.55–0.95	0.78	0.60–1.03
Quintile 3	0.50	0.41–0.60	0.62	0.48–0.81	0.64	0.49–0.83	0.71	0.54–0.93
Quintile 4	0.43	0.35–0.53	0.56	0.43–0.72	0.57	0.44–0.76	0.68	0.51–0.90
Quintile 5	0.35	0.28–0.42	0.52	0.40–0.67	0.55	0.40–0.75	0.71	0.51–0.98
**Women**								
Quintile 1	Reference		Reference		Reference		Reference	
Quintile 2	0.78	0.68–0.89	0.86	0.73–1.01	0.84	0.71–0.99	0.88	0.75–1.04
Quintile 3	0.79	0.69–0.91	0.92	0.78–1.08	0.88	0.74–1.05	0.97	0.81–1.16
Quintile 4	0.73	0.63–0.84	0.83	0.70–0.99	0.80	0.66–0.97	0.90	0.74–1.11
Quintile 5	0.71	0.61–0.83	0.77	0.64–0.93	0.72	0.57–0.91	0.87	0.68–1.12

Model 1 is adjusted for age and sex. Model 2 is adjusted for all variables included in Model 1, diabetes, hypertension, body mass index, smoking status, physical activity, white blood cell count, hemoglobin, and total cholesterol. Model 3 is adjusted for all variables included in Model 2, estimated glomerular filtration rate, and protein intake. Model 4 is adjusted for all variables included in Model 3 and total energy intake. Abbreviations: OR, odds ratio; CI, confidence interval

**Fig. 3:**
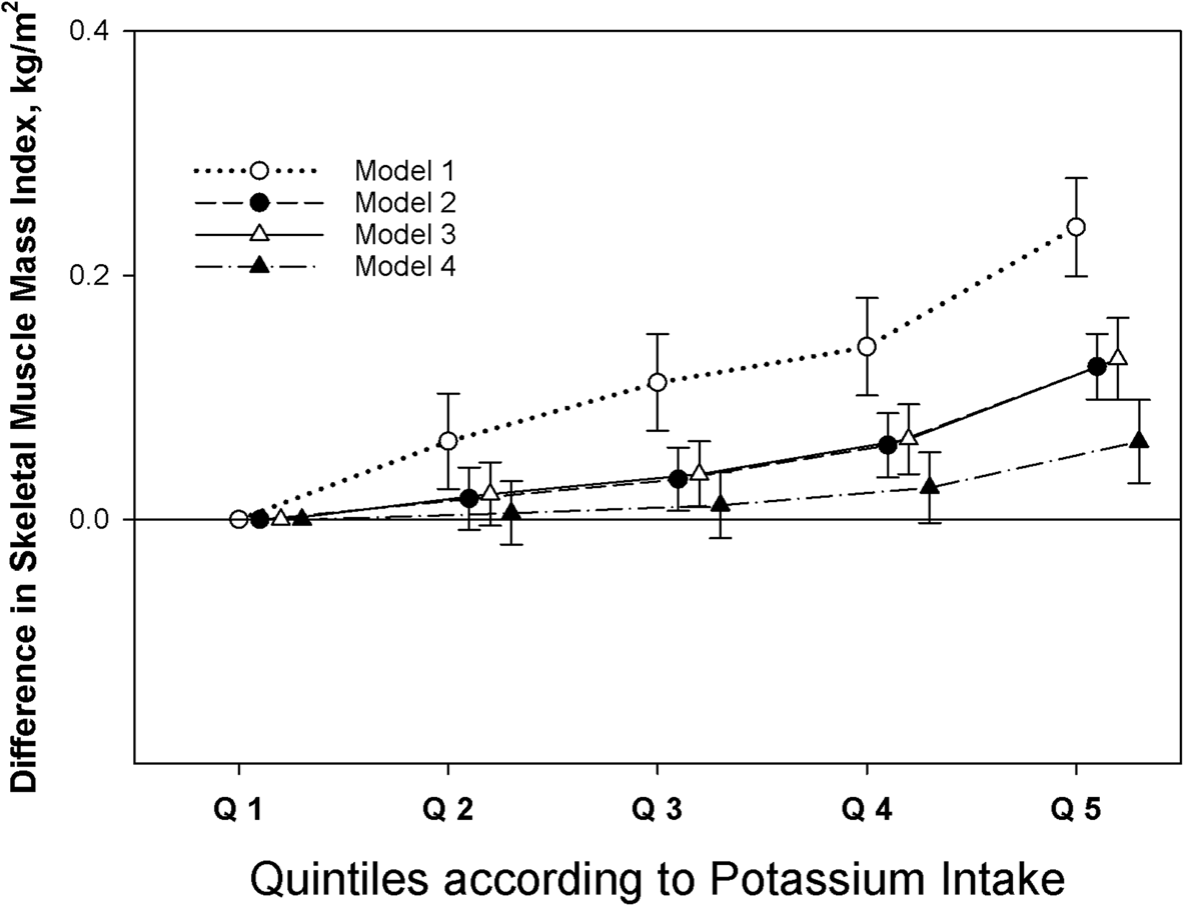
Linear regression analyses with four levels adjustment showing the association between dietary potassium intake and skeletal muscle mass index among 16,558 Korean general population. Model 1 is adjusted for age and sex. Model 2 is adjusted for all variables included in Model 1, diabetes, hypertension, body mass index, smoking status, physical activity, white blood cell count, hemoglobin, and total cholesterol. Model 3 is adjusted for all variables included in Model 2, estimated glomerular filtration rate, and protein intake. Model 4 is adjusted for all variables included in Model 3 and total energy intake

**Fig. 4:**
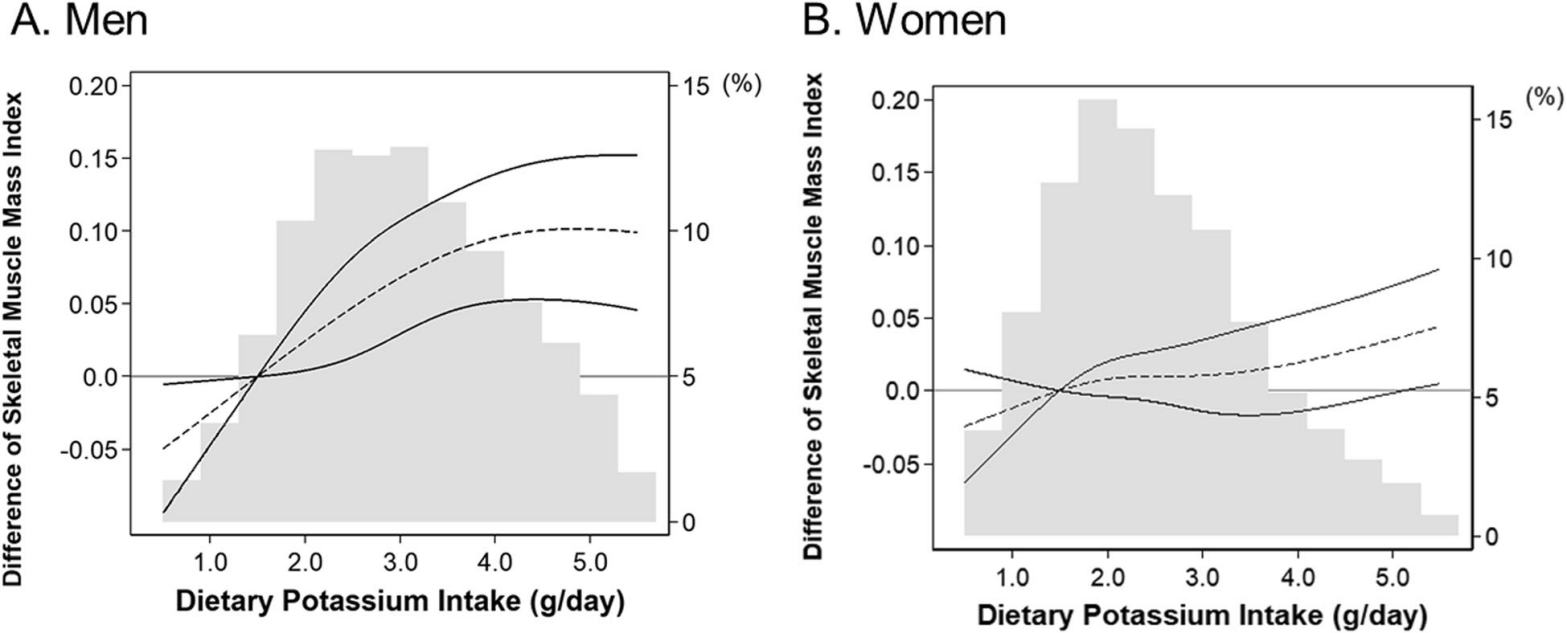
Fully adjusted linear regression model with a restricted cubic spline for the association between dietary potassium intake as a continuous variable and skeletal muscle index according to men (**a**) and women (**b**) among 16,558 Korean general population

In a sensitivity analysis of the association between potassium intake and SMI, higher quintiles of potassium intake were associated with a graded increase of SMI compared to the lowest quintile of potassium intake in all adjusted models, with some attenuation after adjusting for energy intake (Fig. 3). Restricted cubic spline models for the association between potassium intake as a continuous variable and SMI also showed sex-specific differences (Fig. 4a and b).

## Discussion

In this study, we found a trend toward decreased odds for low muscle mass across higher dietary potassium intake in the general population but the association was attenuated after adjusting for total energy intake. Interestingly, sex was observed to be a potential effect modifier of the association between potassium intake and low muscle mass. The association between higher potassium intake and low muscle mass remained robust even after adjusting for energy intake in men but not in women. These findings indicate that dietary potassium intake may affect skeletal muscle mass independent of total energy intake in men but that total energy intake rather than potassium intake per se may affect muscle mass in women.

A previous study showed a favorable effect of alkaline diets on lean tissue mass in adults ≥65 years old by assessing the association between 24-h urinary potassium and the percentage of lean tissue mass [29]. The finding of our study is in line with that of this previous study. The beneficial effects of potassium on skeletal muscle mass may be partially explained by acid-base theory [14, 34]. Chronic metabolic acidosis in chronic kidney disease is associated with protein-energy wasting and loss of muscle mass [14, 35]. Treatment of metabolic acidosis in dialysis patients ameliorates protein-energy wasting [34, 36]. In normal adults, metabolic acidosis also stimulates nitrogen loss due to accelerated degradation of muscle protein [37, 38]. A diet that is high in meats and grains promotes low-grade metabolic acidosis [39]. Treatment with potassium bicarbonate was shown to improve the nitrogen balance in postmenopausal women with mild metabolic acidosis [40]. Therefore, alkaline potassium salts produced from vegetables and fruits may have a protective effect against a loss of muscle mass by neutralizing an acidic condition [41, 42].

Another putative mechanism for the association between potassium intake and muscle mass is their relationship to insulin sensitivity and inflammation. Impaired insulin signaling in cultured muscle cells stimulated proteolysis, causing muscle atrophy [15]. Insulin resistance in patients without diabetes receiving dialysis was associated with skeletal muscle protein breakdown [43]. High potassium intake was associated with a reduced risk for developing insulin resistance or diabetes mellitus [44]. Moreover, chronic inflammation is also a well-known risk factor for loss of muscle mass [45]. Low dietary potassium is known to be pro-inflammatory and associated with increased free radical generation [46]. Therefore, improved insulin resistance and a lower inflammatory state resulting from high potassium intake may help reduce muscle protein breakdown.

The underlying mechanism for the sex-specific difference in the association between potassium intake and muscle mass is unclear. Although the prevalence of sarcopenia was higher for women in that absolute muscle mass was smaller to start with, the rate of loss of muscle mass was higher in men than women in previous longitudinal studies [12]. However, the anabolic response of muscle to nutrition with or without exercise was subtle in older women [47]. Likewise, the benefits of sex hormones such as testosterone on preserving muscle mass also differed by sex; the effects were minimal or unknown in women [12]. Sex-specific differences in muscle protein metabolism, which is thought to be primarily associated with hormone profiles, may exist and may consequently lead to differences in therapeutic effects [48]. Another possible explanation is sex difference in insulin resistance. Insulin resistance is higher in men than women, possibly associated with differences in adipokines and sex hormones [49]. Improved insulin sensitivity in men may benefit more from high potassium intake than in women.

We acknowledge several limitations in our study. First, although we adjusted for potential confounders to investigate the association of dietary potassium intake with low muscle mass, there may be other unmeasured confounding factors. In particular, it might be that adults with higher potassium intakes are those who eat more, are heavier, and have more muscle mass. However, the association between dietary potassium intake and muscle mass remained robust even after adjusting for total energy intake in men. In addition, we could not prove a temporal relationship with our results due to the cross-sectional design of the study. Second, the KNHANES used a 24-h dietary recall method with various measuring aids to assess potassium intake, which might lead to a measurement error. This method, in particular, did not reflect seasonal variations which may underestimate mean potassium intake and attenuate the true association of potassium intake with health outcomes [50]. However, this effect may bias our results toward the null. Furthermore, we used self-reported physical activity. Although the questionnaire was validated, the assessment of physical activity by self-reporting can lead to response bias, thus overestimating actual activity. However, even after the effect of physical activity as a confounding variable was adjusted in our study, the association between primary exposure and outcome was not affected. Third, the mean eGFR in our cohort was 96 mL/min/1.73 m^2^. The proportion of participants with eGFR < 30 mL/min/1.73 m^2^ was only < 0.2%. High potassium intake can cause hyperkalemia and subsequently poor outcomes in patients with decreased renal function. Therefore, the ability to extrapolate our study findings to adults with decreased renal function is limited. Lastly, as data on muscle strength was not available, we could not address whether higher potassium intake was associated with greater muscle strength.

## Conclusions

We demonstrated a positive association of higher potassium intake with lower odds of low muscle mass for men but not for women. Based on our findings, future clinical trials are needed to establish not only a causal relationship between potassium intake and preservation of muscle mass and function, but also an effect modification on that association by sex. Moreover, in further studies, the use of more objective tools for assessment of variables such as potassium intake, muscle mass and function, and physical activity may be more helpful in proving the results of the study.

## Supplementary information

**Additional file 1.** Odds ratios for low muscle mass with four levels of adjustment based upon baseline dietary potassium intake among 16,558 Korean general population. Model 1 is adjusted for age and sex. Model 2 is adjusted for variables included in Model 1, diabetes, hypertension, body mass index, smoking status, physical activity, white blood cell count, hemoglobin, and total cholesterol. Model 3 is adjusted for all variables included in Model 2, estimated glomerular filtration rate, and protein intake. Model 4 is adjusted for all variables included in Model 3 and total energy intake.

## Data Availability

The KNHANES database is publicly available at the KNHANES website (http://knhanes.cdc.go.kr).

## Abbreviations

ASM: Appendicular skeletal muscle mass
BMI: Body mass index
DXA: Dual energy X-ray absorptiometry
eGFR: Estimated glomerular filtration rate
IQR: Interquartile range
ROC: Receiver operating characteristic
sCr: Serum creatinine
SMI: Skeletal muscle index
WBC: White blood cell count
