# Supplementary material for: Potassium intake, skeletal muscle mass, and effect modification by sex: data from the 2008–2011 KNHANES
Source: Nutr J. 2020 Aug 29;19:93. doi: 10.1186/s12937-020-00614-z (PMC7456505; doi:10.1186/s12937-020-00614-z)
Supplement: Supplementary file 1 — Additional file 1. Odds ratios for low muscle mass with four levels of adjustment based upon baseline dietary potassium intake among 16,558 Korean general population. Model 1 is adjusted for age and sex. Model 2 is adjusted for variables included in Model 1, diabetes, hypertension, body mass index, smoking status, physical activity, white blood cell count, hemoglobin, and total cholesterol. Model 3 is adjusted for all variables included in Model 2, estimated glomerular filtration rate, and protein intake. Model 4 is adjusted for all variables included in Model 3 and total energy intake. [file 12937_2020_614_MOESM1_ESM.docx]

**Additional file**

**Additional file 1.** Odds ratios for low muscle mass with four levels of adjustment based upon baseline dietary potassium intake among 16,558 Korean general population. Model 1 is adjusted for age and sex. Model 2 is adjusted for variables included in Model 1, diabetes, hypertension, body mass index, smoking status, physical activity, white blood cell count, hemoglobin, and total cholesterol. Model 3 is adjusted for all variables included in Model 2, estimated glomerular filtration rate, and protein intake. Model 4 is adjusted for all variables included in Model 3 and total energy intake.
